# Supplementary material for: Probiotic Bacillus Strains Enhance T Cell Responses in Chicken
Source: Microorganisms. 2023 Jan 19;11(2):269. doi: 10.3390/microorganisms11020269 (PMC9965164; doi:10.3390/microorganisms11020269)
Supplement: Supplementary file 1 [file microorganisms-11-00269-s001.zip › microorganisms-2142581-supplementary.pdf]

Supplementary Materials:

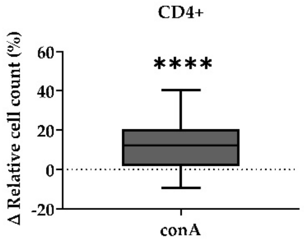

(a)

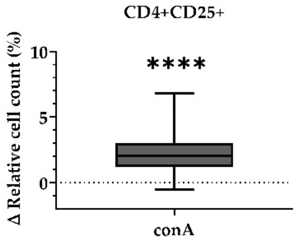

(b)

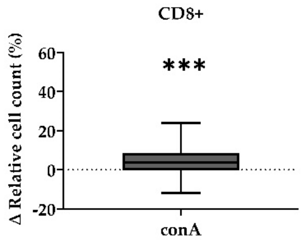

(c)

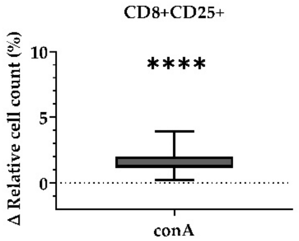

(d)

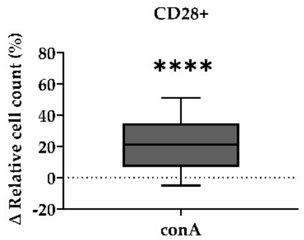

(e)

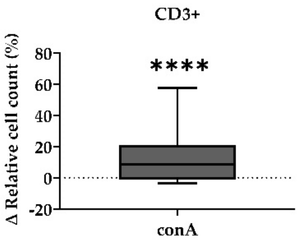

(f)

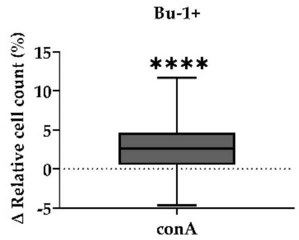

(g)

Figure S1. Influence of conA on activation and proliferation of T cells and B cells. Isolated PBMCs were treated with 10 µg/mL conA as a positive control or left untreated (negative control). After treatment for 24 h, immune cells

were stained with monoclonal antibodies. DAPI was used as a viability marker. a) CD4+ T-helper cells, b) CD4+CD25+ activated T-helper cells, c) CD8+ cytotoxic T cells, d) CD8+CD25+ activated cytotoxic T cells, e) all CD28+ T cells, f) all CD3+ T cells, g) Bu-1+ B cells relative to the vital PBMC cell count. 20,000 vital PBMCs were recorded on a BD FACSCanto II flow cytometer. Data represent the following numbers of biological replicates: 53 (CD4+, CD4+CD25+), 55 (CD8+, CD8+CD25+), 46 (CD28+), and 41 (CD3+, Bu-1+). Results are presented as box and whisker plots showing the median, with 25-75 percentile range as the box and minimum to maximum as the whiskers. Significance is shown as ***, $p < 0.001$; ****, $p < 0.0001$.

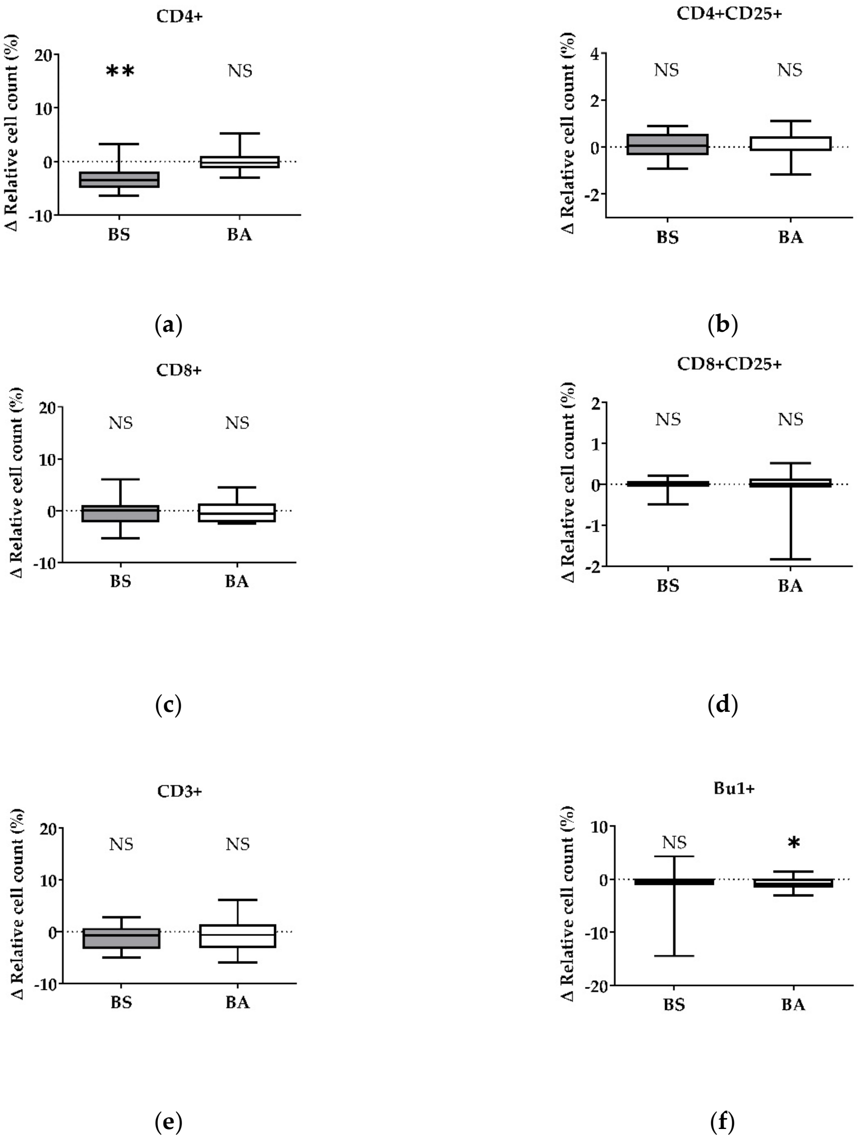

Figure S2. Influence of heat-treated cell-free culture supernatants of *B. subtilis* DSM 32315 (grey) and *B. amyloliquefaciens* CECT 5940 (white) activation and proliferation of T and B cells. Isolated PBMCs were treated with heat-treated cell-free bacterial culture supernatants of BS and BA as a negative control. After treatment for 24 h, immune cells were stained with monoclonal antibodies. DAPI was used as a viability marker. a) CD4+ T-helper cells, b) CD4+CD25+ activated T-helper cells, c) CD8+ cytotoxic T cells, d) CD8+CD25+ activated cytotoxic T cells, e) CD3+ T cells, and f) Bu-1+ B cells relative to the vital PBMC cell count. 20,000 vital PBMCs were recorded on a BD FACSCanto II flow cytometer. Data represent 13 (BS) or 12 (BA) biological replicates.

Results are presented as box and whisker plots showing the median, with 25-75 percentile range as the box and minimum to maximum as the whiskers. Significance is shown as **, $p < 0.01$; *, $p < 0.05$; NS: not significant.

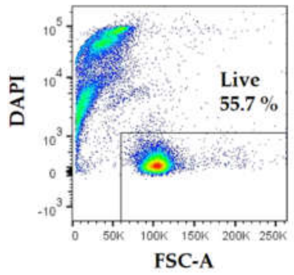

(**a**)

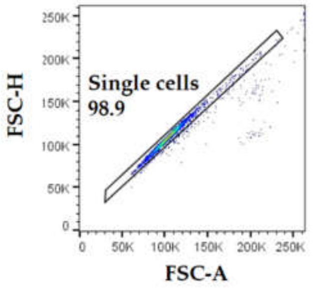

(**b**)

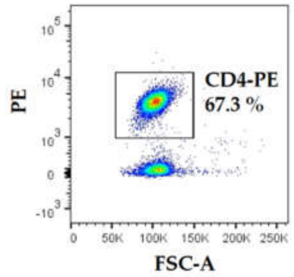

(**c**)

Figure S3. Example of the gating strategy for chicken PBMCs (**a**) y axes: DAPI, DAPI was used as a live/dead marker, x axes: FSC-A, the rectangle gate represents vital PBMC population; (**b**) y axes: FSC-H, x axes: FSC-A, the rectangle gate represents the single cell population out of the vital PBMC population in a), (**c**) y axes: PE, x axes: FSC-A, the rectangle gate represent the CD4-PE T-helper cell population out of the single cell and vital PBMC population in a) and b). Data represent one biological replicate. A total of 20,000 cells were recorded on a BD FACSCanto II flow cytometer.
